# Supplementary material for: Multi‐dataset identification of innovative feature genes and molecular mechanisms in keratoconus
Source: J Cell Mol Med. 2024 Sep 19;28(18):e70079. doi: 10.1111/jcmm.70079 (PMC11412914; doi:10.1111/jcmm.70079)
Supplement: Supplementary file 2 — Material S2. [file JCMM-28-e70079-s002.zip › 7.GSEA analysis/1.ARL11/KEGG_SPLICEOSOME.html]

Details for gene set KEGG\_SPLICEOSOME[GSEA]

|  || Dataset | ARL11.group.cls#h\_versus\_l |
| Phenotype | group.cls#h\_versus\_l |
| Upregulated in class | h |
| GeneSet | KEGG\_SPLICEOSOME |
| Enrichment Score (ES) | 0.17930333 |
| Normalized Enrichment Score (NES) | 0.78191996 |
| Nominal p-value | 0.76254827 |
| FDR q-value | 1.0 |
| FWER p-Value | 1.0 |
Table: GSEA Results Summary

  

Fig 1: Enrichment plot: KEGG\_SPLICEOSOME      
 Profile of the Running ES Score & Positions of GeneSet Members on the Rank Ordered List

  

| SYMBOL | TITLE | RANK IN GENE LIST | RANK METRIC SCORE | RUNNING ES | CORE ENRICHMENT || 1 | THOC1 | na | 4419 | 0.185 | -0.0900 | Yes |
| 2 | HSPA2 | na | 5536 | 0.150 | -0.0916 | Yes |
| 3 | PRPF40A | na | 5760 | 0.144 | -0.0688 | Yes |
| 4 | SART1 | na | 6688 | 0.119 | -0.0714 | Yes |
| 5 | CDC5L | na | 6862 | 0.115 | -0.0531 | Yes |
| 6 | HSPA8 | na | 7386 | 0.104 | -0.0470 | Yes |
| 7 | THOC2 | na | 7570 | 0.100 | -0.0319 | Yes |
| 8 | RBM25 | na | 7934 | 0.094 | -0.0233 | Yes |
| 9 | SRSF1 | na | 8396 | 0.085 | -0.0193 | Yes |
| 10 | SF3B3 | na | 8566 | 0.082 | -0.0076 | Yes |
| 11 | ACIN1 | na | 8639 | 0.080 | 0.0067 | Yes |
| 12 | SRSF6 | na | 9024 | 0.074 | 0.0107 | Yes |
| 13 | NCBP1 | na | 9152 | 0.073 | 0.0218 | Yes |
| 14 | HSPA1A | na | 9156 | 0.072 | 0.0365 | Yes |
| 15 | HSPA6 | na | 9438 | 0.068 | 0.0422 | Yes |
| 16 | SRSF5 | na | 10103 | 0.059 | 0.0350 | Yes |
| 17 | PRPF19 | na | 10399 | 0.055 | 0.0377 | Yes |
| 18 | HNRNPA1L2 | na | 10562 | 0.053 | 0.0438 | Yes |
| 19 | HSPA1B | na | 10821 | 0.050 | 0.0465 | Yes |
| 20 | MAGOHB | na | 10854 | 0.049 | 0.0555 | Yes |
| 21 | SRSF3 | na | 11041 | 0.047 | 0.0598 | Yes |
| 22 | SRSF8 | na | 11213 | 0.045 | 0.0640 | Yes |
| 23 | DDX46 | na | 11816 | 0.038 | 0.0543 | Yes |
| 24 | XAB2 | na | 11958 | 0.037 | 0.0577 | Yes |
| 25 | PHF5A | na | 12312 | 0.033 | 0.0542 | Yes |
| 26 | CDC40 | na | 12315 | 0.033 | 0.0609 | Yes |
| 27 | ISY1 | na | 12539 | 0.031 | 0.0608 | Yes |
| 28 | HNRNPA1 | na | 12555 | 0.031 | 0.0667 | Yes |
| 29 | HNRNPU | na | 12785 | 0.029 | 0.0659 | Yes |
| 30 | SRSF2 | na | 12856 | 0.028 | 0.0696 | Yes |
| 31 | EFTUD2 | na | 12889 | 0.028 | 0.0744 | Yes |
| 32 | U2AF2 | na | 12949 | 0.027 | 0.0783 | Yes |
| 33 | CRNKL1 | na | 13077 | 0.026 | 0.0800 | Yes |
| 34 | LSM3 | na | 13318 | 0.024 | 0.0780 | Yes |
| 35 | U2SURP | na | 13992 | 0.019 | 0.0624 | Yes |
| 36 | U2AF1 | na | 14111 | 0.018 | 0.0626 | Yes |
| 37 | DHX8 | na | 14201 | 0.017 | 0.0635 | Yes |
| 38 | PRPF40B | na | 14486 | 0.015 | 0.0584 | Yes |
| 39 | RBM22 | na | 14586 | 0.014 | 0.0585 | Yes |
| 40 | HNRNPK | na | 14597 | 0.014 | 0.0611 | Yes |
| 41 | PRPF8 | na | 14927 | 0.012 | 0.0539 | Yes |
| 42 | TRA2B | na | 15024 | 0.011 | 0.0534 | Yes |
| 43 | LSM7 | na | 15215 | 0.010 | 0.0499 | Yes |
| 44 | SNRNP200 | na | 15539 | 0.007 | 0.0420 | Yes |
| 45 | NCBP2 | na | 15628 | 0.006 | 0.0408 | Yes |
| 46 | PRPF4 | na | 15700 | 0.006 | 0.0399 | Yes |
| 47 | SF3B1 | na | 15735 | 0.006 | 0.0401 | Yes |
| 48 | PLRG1 | na | 15897 | 0.004 | 0.0363 | Yes |
| 49 | RBM17 | na | 15966 | 0.004 | 0.0351 | Yes |
| 50 | DDX39B | na | 16095 | 0.003 | 0.0321 | Yes |
| 51 | SNRPD1 | na | 16265 | 0.002 | 0.0276 | Yes |
| 52 | SNRPD2 | na | 16268 | 0.002 | 0.0279 | Yes |
| 53 | MAGOH | na | 16332 | 0.001 | 0.0264 | Yes |
| 54 | SRSF10 | na | 16417 | 0.001 | 0.0241 | Yes |
| 55 | TRA2A | na | 16603 | -0.001 | 0.0189 | Yes |
| 56 | SNRPF | na | 16604 | -0.001 | 0.0190 | Yes |
| 57 | PRPF6 | na | 16822 | -0.002 | 0.0131 | Yes |
| 58 | HSPA1L | na | 16824 | -0.002 | 0.0135 | Yes |
| 59 | ALYREF | na | 16929 | -0.003 | 0.0111 | Yes |
| 60 | HNRNPM | na | 16996 | -0.003 | 0.0098 | Yes |
| 61 | DDX5 | na | 17025 | -0.003 | 0.0097 | Yes |
| 62 | CCDC12 | na | 17300 | -0.005 | 0.0029 | Yes |
| 63 | SNRPE | na | 17377 | -0.006 | 0.0019 | Yes |
| 64 | SNRNP40 | na | 17594 | -0.007 | -0.0028 | Yes |
| 65 | HNRNPA3 | na | 17668 | -0.008 | -0.0033 | Yes |
| 66 | SF3A1 | na | 17669 | -0.008 | -0.0017 | Yes |
| 67 | SRSF9 | na | 17818 | -0.009 | -0.0041 | Yes |
| 68 | AQR | na | 17824 | -0.009 | -0.0024 | Yes |
| 69 | SF3A2 | na | 17893 | -0.010 | -0.0024 | Yes |
| 70 | DDX23 | na | 18036 | -0.010 | -0.0044 | Yes |
| 71 | ZMAT2 | na | 18175 | -0.012 | -0.0061 | Yes |
| 72 | SNRPG | na | 18255 | -0.012 | -0.0059 | Yes |
| 73 | RBM8A | na | 18310 | -0.012 | -0.0049 | Yes |
| 74 | PPIL1 | na | 18316 | -0.012 | -0.0025 | Yes |
| 75 | CWC15 | na | 18501 | -0.014 | -0.0051 | Yes |
| 76 | SRSF4 | na | 18565 | -0.014 | -0.0040 | Yes |
| 77 | SF3B5 | na | 18669 | -0.015 | -0.0040 | Yes |
| 78 | SF3B2 | na | 19166 | -0.018 | -0.0147 | Yes |
| 79 | USP39 | na | 19434 | -0.020 | -0.0183 | Yes |
| 80 | SNRPA | na | 19452 | -0.020 | -0.0147 | Yes |
| 81 | LSM8 | na | 19493 | -0.020 | -0.0117 | Yes |
| 82 | CTNNBL1 | na | 19821 | -0.023 | -0.0166 | Yes |
| 83 | TXNL4A | na | 19987 | -0.024 | -0.0166 | Yes |
| 84 | CHERP | na | 20090 | -0.024 | -0.0146 | Yes |
| 85 | LSM5 | na | 20293 | -0.026 | -0.0151 | Yes |
| 86 | SF3B6 | na | 20497 | -0.027 | -0.0154 | Yes |
| 87 | SF3A3 | na | 20737 | -0.029 | -0.0164 | Yes |
| 88 | PCBP1 | na | 20857 | -0.030 | -0.0136 | Yes |
| 89 | BUD31 | na | 20950 | -0.031 | -0.0100 | Yes |
| 90 | PRPF38A | na | 20963 | -0.031 | -0.0041 | Yes |
| 91 | SNRPB2 | na | 21015 | -0.031 | 0.0008 | Yes |
| 92 | PRPF31 | na | 21040 | -0.032 | 0.0066 | Yes |
| 93 | WBP11 | na | 21267 | -0.033 | 0.0068 | Yes |
| 94 | PRPF18 | na | 21317 | -0.034 | 0.0123 | Yes |
| 95 | HNRNPC | na | 21434 | -0.035 | 0.0160 | Yes |
| 96 | LSM4 | na | 21469 | -0.035 | 0.0221 | Yes |
| 97 | THOC3 | na | 21535 | -0.036 | 0.0275 | Yes |
| 98 | SNW1 | na | 21604 | -0.036 | 0.0328 | Yes |
| 99 | SNRPD3 | na | 21765 | -0.037 | 0.0358 | Yes |
| 100 | LSM2 | na | 22090 | -0.041 | 0.0348 | Yes |
| 101 | PUF60 | na | 22102 | -0.041 | 0.0428 | Yes |
| 102 | SNRNP70 | na | 22137 | -0.041 | 0.0502 | Yes |
| 103 | EIF4A3 | na | 22261 | -0.043 | 0.0553 | Yes |
| 104 | PRPF38B | na | 22262 | -0.043 | 0.0639 | Yes |
| 105 | DDX42 | na | 22306 | -0.043 | 0.0714 | Yes |
| 106 | SLU7 | na | 22383 | -0.044 | 0.0781 | Yes |
| 107 | RBMX | na | 22535 | -0.045 | 0.0830 | Yes |
| 108 | PQBP1 | na | 22698 | -0.047 | 0.0879 | Yes |
| 109 | PPIE | na | 22719 | -0.047 | 0.0970 | Yes |
| 110 | DHX15 | na | 22808 | -0.048 | 0.1042 | Yes |
| 111 | DHX16 | na | 22832 | -0.048 | 0.1134 | Yes |
| 112 | DHX38 | na | 22932 | -0.049 | 0.1205 | Yes |
| 113 | BCAS2 | na | 23170 | -0.052 | 0.1243 | Yes |
| 114 | PPIH | na | 23173 | -0.052 | 0.1348 | Yes |
| 115 | SNRPB | na | 23281 | -0.053 | 0.1425 | Yes |
| 116 | LSM6 | na | 23466 | -0.055 | 0.1485 | Yes |
| 117 | SNRPA1 | na | 23721 | -0.059 | 0.1531 | Yes |
| 118 | SNRPC | na | 23767 | -0.059 | 0.1639 | Yes |
| 119 | TCERG1 | na | 24289 | -0.066 | 0.1623 | Yes |
| 120 | SNRNP27 | na | 24672 | -0.072 | 0.1659 | Yes |
| 121 | PRPF3 | na | 24797 | -0.074 | 0.1772 | Yes |
| 122 | SRSF7 | na | 25973 | -0.090 | 0.1616 | Yes |
| 123 | SMNDC1 | na | 25995 | -0.090 | 0.1793 | Yes |
| 124 | SYF2 | na | 28535 | -0.144 | 0.1353 | No |
| 125 | SF3B4 | na | 30815 | -0.214 | 0.1131 | No |
Table: GSEA details [plain text format]

  

Fig 2: KEGG\_SPLICEOSOME      
 Blue-Pink O' Gram in the Space of the Analyzed GeneSet

  

Fig 3: KEGG\_SPLICEOSOME: Random ES distribution      
 Gene set null distribution of ES for **KEGG\_SPLICEOSOME**
